# Inhibition of EZH2 via activation of SAPK/JNK and reduction of p65 and DNMT1 as a novel mechanism in inhibition of human lung cancer cells by polyphyllin I

**DOI:** 10.1186/s13046-016-0388-x

**Published:** 2016-07-16

**Authors:** Longmei Li, JingJing Wu, Fang Zheng, Qing Tang, WanYin Wu, Swei Sunny Hann

**Affiliations:** Laboratory of Tumor Molecular Biology and Targeted Therapies of TCM, Guangdong Provincial Hospital of Chinese Medicine, No. 111, Dade Road, Guangzhou, Guangdong Province 510120 China; Department of Medical Oncology, Guangdong Provincial Hospital of Chinese Medicine, The Second Clinical Medical Collage, University of Guangzhou Traditional Chinese Medicine, Guangzhou, Guangdong Province 510120 China

**Keywords:** PPI, NSCLC, SAPK/JNK, NF-kB/p65, EZH2, DNMT1

## Abstract

**Background:**

Polyphyllin I (PPI), a bioactive phytochemical extracted from the Rhizoma of *Paris polyphylla*, has been reported to exhibit anti-cancer activity. However, the detailed mechanism underlying this remains to be elucidated.

**Methods:**

Cell viability and cell cycle distribution were measured using a 3-(4, 5-dimethylthiazol-2-yl)-2, 5-diphenyltetrazolium bromide (MTT) and flow cytometry assays, respectively. The expression of enhancer of zeste homolog 2 (EZH2) mRNA was measured by quantitative real time PCR (qRT-PCR). Western blot analysis was performed to examine the phosphorylation and protein expression of stress-activated protein kinase/c-Jun N-terminal kinase (SAPK/JNK), p65, DNA methyltransferase 1 (DNMT1) and EZH2. Exogenous expression of p65, DNMT1, and EZH2 were carried out by transient transfection assays. Promoter activity of EZH2 gene was determined using Secrete-Pair Dual Luminescence Assay Kit. A xenografted tumor model in nude mice and bioluminescent imaging system were used to further test the effect of PPI in vivo.

**Results:**

We showed that PPI significantly inhibited growth and induced cell cycle arrest of non-small cell lung cancer (NSCLC) cells in a dose-dependent manner. Mechanistically, we found that PPI increased the phosphorylation of SAPK/JNK, reduced protein expression of p65 and DNMT1. The inhibitor of SAPK/JNK (SP600125) blocked the PPI-inhibited p65 and DNMT1 protein expression. Interestingly, exogenously expressed p65 overcame PPI-inhibited protein expression of DNMT1. Moreover, PPI reduced EZH2 protein, mRNA, and promoter activity; overexpression of EZH2 resisted the PPI-inhibited cell growth, and intriguingly, negative feedback regulation of SAPK/JNK signaling. Finally, exogenous expression of DNMT1 antagonized the PPI-suppressed EZH2 protein expression. Consistent with this, PPI inhibited tumor growth, protein expression levels of p65, DNMT1 and EZH2, and increased phosphorylation of SAPK/JNK in vivo*.*

**Conclusion:**

Our results show that PPI inhibits growth of NSCLC cells through SAPK/JNK-mediated inhibition of p65 and DNMT1 protein levels, subsequently; this results in the reduction of EZH2 gene expression. The interactions among p65, DNMT1 and EZH2, and feedback regulation of SAPK/JNK by EZH2 converge on the overall responses of PPI. This study reveals a novel mechanism for regulating EZH2 gene in response to PPI and suggests a new strategy for NSCLC associated therapy.

## Background

Lung cancer is the most common type of malignancy worldwide, and the leading cancer-related cause of death of both men and women. The majority of lung cancers are the non-small cell lung cancer (NSCLC) presented with advanced stage [1]. Despite recent advances in clinical and experimental studies in the treatment of lung cancer, the prognosis still remains poor due to the uncountable recurrence and metastasis [1, 2]. Therefore, there is an urgent need to search new agents with minimal side effects and improved treatment efficacy. These included components from Chinese medical herbal plants; among those, polyphyllin I (PPI) may be one of such candidates.

PPI, a bioactive phytochemical extracted from the Rhizoma of *Paris polyphylla*, has been reported to possess preclinical anticancer efficacy in various cancer types [3–6]. PPI induced apoptosis and reversed epithelial mesenchymal transition in human osteosarcoma cells [7]. Also, PPI triggered cell apoptosis, and inhibited cell growth via regulating caspase activation pathway, increasing c-Jun expression and reducing differential gene, such as phosphatidylinositol-4-phosphate 3-kinase catalytic subunit type 2 beta (PIK3C2β) and wingless-type MMTV integration site family member 5A (Wnt5A), expressions in human ovarian cancer cells [5]. Moreover, one study showed that PPI exhibited anti-tumor effect in NSCLC cells in vitro and in vivo at least through induction of apoptotic signaling [8]. Nevertheless, the underlying molecular mechanisms in targeting lung cancer remain largely unknown.

Nuclear factor-kappaB (NF-kB), a key transcription factor, is involved in critical mechanisms connecting to inflammation, cancer occurrence, and progression, among others. NF-kB is activated by a variety of extracellular signals, and regulates the expression of a variety of genes [9]. The constitutive NF-kB activity was found in a larger numbers of human cancers due to the inflammatory microenvironment, various oncogenic mutations and inactivation of tumor suppressors. Given the most likely tumor promoting role, targeting NF-kB could be beneficial in the prevention and treatment of various types of tumors including lung cancer [10–13]. Surprisingly, until now there are no studies demonstrating the potential role of NF-kB and its downstream signaling in mediating the therapeutic effects of PPI. Therefore, the detailed function and relevant mechanism of this transcription factor involving in the anti-cancer response of PPI remains unknown.

DNA methyltransferases (DNMTs) catalyze the methylation at cytosine-C5 mainly in a CpG dinucleotide context. Among the four active members (DNMT1, DNMT3A, DNMT3B, and DNMT3L), DNMT1 is the most abundant one responsible for maintenance of the DNA methylation pattern. However, the exact mechanism of suppression of DNMT1 signaling is not elucidated. Likely mechanisms include enzymatic inhibition, reduced DNMT1 expression [14]. Overexpression of DNMT1 has been shown in several cancers including lung [15–18]. Inhibition of DNMTs reduced tumor formation, at least in part through the increased expression of tumor suppressor gene [19]. Thus, targeting DNMT1 could be a potential in the prevention and treatment of cancers [20–23].

Enhancer of zeste homologue 2 (EZH2), a potential epigenetic silencer of tumor suppressor genes, is frequently highly expressed in a wide variety of human cancers [24]. Previous studies have showed that expression of EZH2 was correlated with proliferation, differentiation, progression and metastasis in several cancer types including lung. Therefore, inhibition of EZH2 through direct and indirect mechanisms, such as epigenetic activation of oncogenic signaling cascades and silencing of tumor suppressor genes, could be an important approach for cancer treatment [21, 25–28]. Moreover, the mechanistically interaction between EZH2 and DNMT1 regulated their downstream targets, thereby controlling DNA methylation and/or transcriptional repressed micoRNAs (miRNAs) expression, which contributed to growth and progression in several cancer types [29, 30].

In this study, we demonstrated the inhibitory effect of PPI on lung cancer cell growth through stress-activated protein kinase/c-Jun N-terminal kinase (SAPK/JNK)-mediated inhibition of NF-kB subunit p65 and DNMT1 protein levels, subsequently; this resulted in the reduction of EZH2 gene expression.

## Methods

### Reagents and cell culture

Monoclonal antibodies specific to total SAPK/JNK and the phosphor-form (thr183/tyr185), EZH2, DNMT1, NF-kB/p65, p50 and SP600125 (JNK inhibitor) were purchased from Cell Signaling Technology Inc. (Beverly, MA, USA). Lipofectamine 3000 reagent was ordered from Life Technologies (AB & Invitrogen, Carlsbad, CA, USA). PPI was purchased from Chengdu Must Biotechnology (Chengdu, China). The 3-(4, 5-dimethylthiazol-2-yl)-2, 5-diphenyltetrazolium bromide (MTT) power was purchased from Sigma Aldrich (St. Louis, Mo, USA). Cell Cycle Staining Kit was obtained from MultiSciences Biotech Co. (Hangzhou, China). Geneticin (G-418 Sulfate) was obtained from Life Technologies (Grand Island, NY, USA). The D-luciferin was obtained from PerkinElmer (Waltham, MA, USA). PC9, A549 NSCLC cells were obtained from the Chinese Academy of Sciences Cell Bank of Type Culture Collection (Shanghai, China). The cells were cultured at 37 ° C in a humidified atmosphere containing 5 % CO2. The culture medium consisted of RPMI 1640 medium obtained from GIBCO, Life Technologies (Grand Island, NY, USA) supplemented with 10 % (v/v) heat-inactivated fetal bovine serum (Thermo Fisher Scientific Inc, Waltham, MA, USA), 100 μg/ml streptomycin and 100 U/mL penicillin. The medium of A549-luc was added G-418 sulfate at concentration of 300 μg/mL. When cells reached 75 % confluence, they were digested with 0.25 % trypsin for passage for the following experiments. The PPI was dissolved in a small amount of dimethylsufoxide [DMSO, maximum concentration, 0.1 % (v/v)], which was then added to complete cell culture medium. Cells treated with vehicle only (DMSO, 0.1 % in media) was served as control.

### Cell viability assay

Cell viability was measured using the 3-(4, 5-dimethylthiazol-2-yl)-2, 5-diphenyltetrazolium bromide (MTT) method as described previously [31]. Briefly, the cells were seeded into a 96-well plate at 5 × 10^3^ cells/well, and incubated at 37 °C with 5 % CO_2_ for 24 h before treated with increasing concentrations of PPI for up to 72 h. In separate experiments, NSCLC cells were transfected with control, pCMV6-EZH2 for 24 h before exposure of the cells to PPI for an additional 24 h. Afterwards, absorbance at 570 nm was determined through the use of ELISA reader (Perkin Elmer, Victor X5, Waltham, MA, USA). Cell viability (%) was calculated as follows: (absorbance of test sample/absorbance of control) × 100 %.

### Cell cycle analysis

This procedure was reported previously [32]. In brief, PC9 cells were cultured in 6-well culture plates at 3 × 10^5^ cells/well and treated with increased doses of PPI for 24 h. Afterwards, the cells were washed and resuspended in PBS and incubated with 0.1 % sodium citrate containing propidium iodide (PI) 0.05 mg and 50 μg RNase for 1 h at room temperature (RT). The cells were washed and subjected to FACScalibur flow cytometric analysis (FC500, Beckman Coulter, FL, USA), and the proportion of cells within the G0/G1, S, and G2/M phases of the cell cycle were analyzed using the MultiCycle AV DNA Analysis software (Phoenix Flow Systems, Inc. San Diego, CA, USA).

### Invasion assay

The invasion ability was detected by the Transwell plate (Corning, NY, USA) with 10 mm diameter and 8 μM pore size polycarbonate membrane. In brief, the matrigel (BD Bioscience, San Jose, CA, USA) was diluted to 8 folds, which was injected into the upper chamber before the experiment. The lower chamber was added with 500 μL of cell culture medium with 20 % FBS. Afterward, A549 and PC9 cells were diluted to 0.5 × 10^6^/mL and pretreated with PPI (0.8 μM), and added 200 μL cells suspension into the upper chamber, then incubated cells at 37 °C in a 5 % CO_2_ for 16 h. Non-migration cells were removed and invaded cells were fixed with 4 % paraformaldehyde, and stained with crystal violet. Absorbance at 570 nm was determined through the use of ELISA reader (Perkin Elmer, Victor × 5, Waltham, MA, USA). Pictures were taken under 100× magnification and the number of invaded cells was counted as percentage of control.

### Transient transfection assays

The detailed procedure was reported previously [33]. Briefly, NSCLC cells were seeded at a density of 2.5 × 10^5^ cells/well in 6-well dishes and grown to 60 % confluence. For each well, 2 μg of the control or wild type pEZX-PG04-EZH2 promoter constructs (purchased from GeneCopoeia, Inc., Rockville, MD, USA) with or without 0.2 μg of the internal control secreted alkaline phosphatase (SEAP) were co-transfected into the cells with the EndoFectin Max transfection reagent. The preparation of cell extracts and measurement of luciferase activities were determined using the Secrete-Pair Dual Luminescence Assay Kit (GeneCopoeia, Inc., Rockville, MD, USA), and was normalized with SEAP activity within each sample. In the separated experiment, 2 μg of the control (pCMV6) and expression constructs containing Myc-DDK-tagged- or Myc/FLAG-tagged ORFs of human DNMT1 or EZH2 obtained from OriGene Technologies, Inc. (Rockville, MD, USA), the control (pCMV4) and p65 overexpression vector (pCMV4-p65) obtained from the Addgene (Plasmid #21966, Cambridge, MA, USA) [34] at a final concentration of 2 μg/mL, were transfected into the cells with the Lipofectamine 3000 reagent. Cells were incubated for 24 h at 37 °C, then treated with PPI for the indicated time for all other experiments.

### Quantitative real-time PCR (qRT-PCR)

A quantitative real-time-PCR (qRT-PCR) assay was developed for the detection of EZH2 transcript. The primers used in this study were designed as follows: EZH2 forward 5′- AATCAGAGTACATGCGACTGAGA -3′; reverse 5′- GCTGTATCCTTCGCTGTTTCC -3′; GAPDH forward 5′- AAGCCTGCCGGTGACTAAC -3′; reversed 5′- GCGCCCAATACGACCAAATC -3′. qRT-PCR was performed in a 20 μL mixture containing 2 μL of the cDNA preparation, 10 μL 2 × SYBR Green Premix ExTaq, and 10 μM primer on an ABI 7500 Real-Time PCR System (Applied Biosystems, Grand Island, NY, USA). The PCR conditions were as follows: 10 min at 95 °C, followed by 40 cycles of 15 s at 95 °C, and 1 min at 60 °C. Each sample was tested in triplicate. Threshold values were determined for each sample/primer pair, the average and standard errors were calculated. The relative expression levels of EZH2 were normalized to that of GAPDH. The data were analyzed using the comparative threshold cycle (2^−ΔΔCT^) method.

### Western blot analysis

The detailed method was based on previous report [31]. The cell lysates containing same amount of protein were separated on SDS polyacrylamide gels. Membranes (Millipore, Billerica, MA, USA) were incubated with antibodies against the total and phosphor-form SAPK/JNK, p50, p65, EZH2 and DNMT1 (1:1000). The membranes were washed and incubated with a secondary antibody raised against rabbit IgG conjugated to horseradish peroxidase (Cell Signaling, Beverly, MA, USA), followed by transferring to freshly made ECL solution (Immobilon Western; Millipore, Billerica, MA, USA), and documented the signals using the Gel Imagine System (Bio-Rad, Hercules, CA, USA).

### Xenograft tumors and bioluminescent imaging

The experiments were performed according to the guidelines for the care and use of laboratory animals approved by the Animal Care and Use Committee of Guangdong Provincial Hospital of Chinese Medicine (the Ethics Approval Number 2014014) and the National Institutes of Health guide for the care and use of Laboratory animals. A total of 36 eight-week-old female nude mice obtained from Guangdong Provincial Research Center for Laboratory Animal Medicine (Foshan, Guangdong, China) were maintained at the Animal Center of Guangdong Provincial Hospital of Chinese Medicine in a specific pathogen-free environment with food and water provided. A549 NSCLC cells carrying luciferase report gene (A549-Luc, obtained from the Guangzhou Land Technology Co., Guangzhou, China) (1 × 10^6^ cells) in 100 μL PBS were injected subcutaneously in the upper hind limb of mice. Xenografts were allowed to grow for over one week when the initial measurement was made with calipers. Mice were randomly divided into control, low (1 mg/kg) and high doses (3 mg/kg) of PPI group, which given once a day by intraperitoneal injection for up to 27 days (*n* = 12/group).

For bioluminescence imaging (BLI) procedure, mice were anesthetized by inhalation of 2 % isoflurane. The substrate D-luciferin (Caliper Life Sciences, Hopkinton, MA, USA) was injected into the peritoneal cavity with a dose of 150 mg/kg in approximately 100 μL. The intensity of BLI signal was determined using the IVIS-200 Imaging System (Xenogen/Caliper, Alameda, CA, USA). Tumor volume measurements were calculated using the formula for an oblong sphere: volume = (width^2^ × length). Quantification of bioluminescence was reported as photons/sec. The body weights of the mice were measured once a week. All mice were euthanized on day 27 in accordance with the Guide for the Care and Use of Laboratory Animals.

### Immunohistochemistry procedures

Immunoperoxidase staining for p-SAPK/JNK, DNMT1, p65, EZH2 proteins were performed on all tissues from representative subjects (control, PPI-treated groups). Briefly, the antigen was retrieved by citric acid buffer (pH 6.0) and then immersed in 3 % H_2_O_2_ to inhibit endogenous peroxidase activity, followed by incubation in 5 % bovine serum albumin to block nonspecific binding. The sections were then incubated with primary antibodies against p-SAPK/JNK (1:100), p65 (1:100), EZH2 (1:50), DNMT1 (1:100) at 4 °C overnight and then incubated with biotinylated secondary antibody (Beijing Zhongshan Golden Bridge Biotechnology Co., Ltd. Beijing, China) for 10 min. Detection was made using the 3,3 -diaminobenzidine according to the instructions (DAB kit, Beijing Zhongshan Golden Bridge Biotechnology Co., Ltd. China). Pictures were taken under 200× magnification. The immunostaining was evaluated by Image-Pro Plus6.0 image analysis software (Media Cybernetics, lnc. Sliver Spring, MD, USA) in at least five random high-power fields.

### Statistical analysis

All data were expressed as mean ± SD of three independent experiments. Differences between groups were assessed by one-way ANOVA and significance of difference between particular treatment groups was analysed by Tukey’s Multiple Comparison Test for multiple comparison involved using GraphPad Prism software version 5.0 (GraphPad Software, Inc. La Jolla, CA, USA). The results in the most graphs were presented relative to the control. Asterisks showed in the figures indicated significant differences of experimental groups in comparison with the corresponding control condition. *P*-values <0.05 were considered statistically significant.

## Results

### PPI inhibited growth and induced cell cycle arrest of human NSCLC cells

**Fig. 1:**
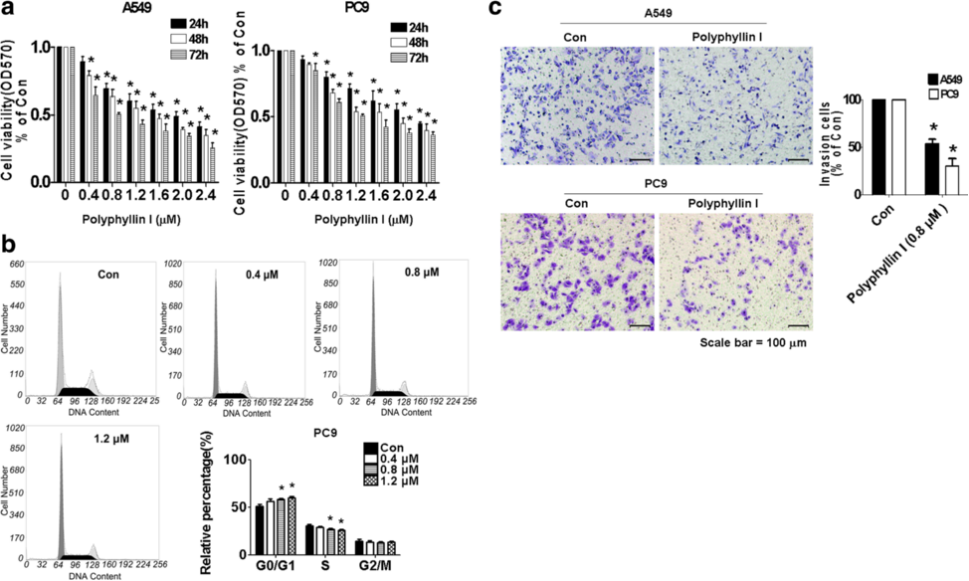
PPI inhibited growth and induced cell cycle arrest of human NSCLC cells. **a** Lung cancer cell lines (PC9 and A549) were treated with increased concentrations of polyphyllin (PPI) for up to 72 h. Afterwards, the cell viability was determined using the MTT assay as described in the Materials and Methods section. **b** PC9 cells were treated with increased concentrations of PPI for up to 48 h. Afterwards, the cells were collected and processed for analysis of cell cycle distribution by flow cytometry. The percentages of the cell population in each phase (G0/G1, S and G2/M) were assessed by Multicycle AV DNA Analysis Software. **c** PC9 and A549 cells were treated with 0.8 μM PPI for up to 24 h. Shown are representative images of fixed and crystal violet-stained PC9 and A549 cell invasion on the matrigel-coated inserts in the presence of vehicle (Con), and polyphyllin (PPI). Values are given as the mean ± SD from 3 independent experiments and expressed as a percentage of total cells. Scale bar 100 μM. *Indicates significant difference as compared to the untreated control group (*P* < 0.05). **Indicates significant difference from PPI treated alone (*P* < 0.05)

We first set up to detect the effect of PPI on cell growth in human NSCLC cells A549 and PC9 by MTT assay. As show in Fig. 1a, PPI decreased the cell viability in a dose-dependent manner with effective dose ranges of 0.8–1.2 μM for up to 72 h treatment. The IC 50s were 1.78, 1.385 and 0.84 μM in A549 and 2.19, 1.64 and 1.32 μM in PC9, respectively.

We also examined the effect of PPI on cell cycle phase distribution. We found that PC9 cells treated with increased concentrations of PPI for 24 h resulted in increase in the proportion of cells at G0/G1 phase, while the proportion of cells at S phases were reduced (Fig. 1b) as detected by flow cytometry. The above results indicated that PPI induced cell cycle arrest at G0/G1 phase in NSCLC cells. Finally, cell invasion assays showed that PPI decreased the ability of invasion compared to the control untreated one in A549 and PC9 cells (Fig. 1c).

### PPI increased phosphorylation of SAPK/JNK in a time-dependent manner

**Fig. 2:**
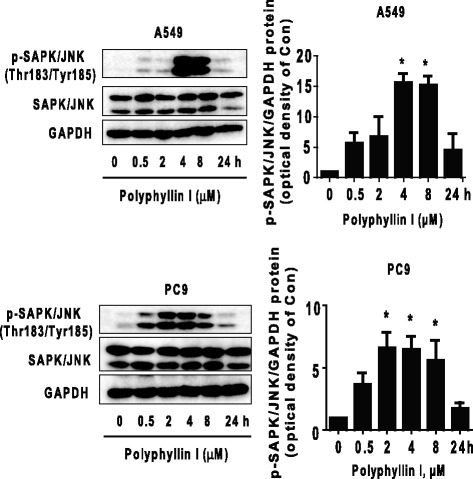
PPI increased phosphorylation of SAPK/JNK in a time-dependent manner. A549 and PC9 cells were treated with PPI (1.6 μM) in the indicated times, and cell lysate was harvested and the expression of phosphorylated or total protein of SAPK/JNK were measured by Western blot. GAPDH was used as loading control. Values in bar graphs were given as the mean ± SD from three independent experiments. *Indicates significant difference as compared to the untreated control group (*P* < 0.05)

SAPK/JNK signaling pathway was involved in cell growth depending on the cell types and stimulus. We showed that PPI increased phosphorylation of SAPK/JNK starting at 0.5 and 4 h, and continuing for up to 4 and 8 h in PC9 and A549 cells, respectively (Fig. 2). Note that the expression of total SAPK/JNK proteins had no significant changes after PPI treatment.

### PPI decreased protein expression levels of DNMT1 and EZH2 through SAPK/JNK pathway

**Fig. 3:**
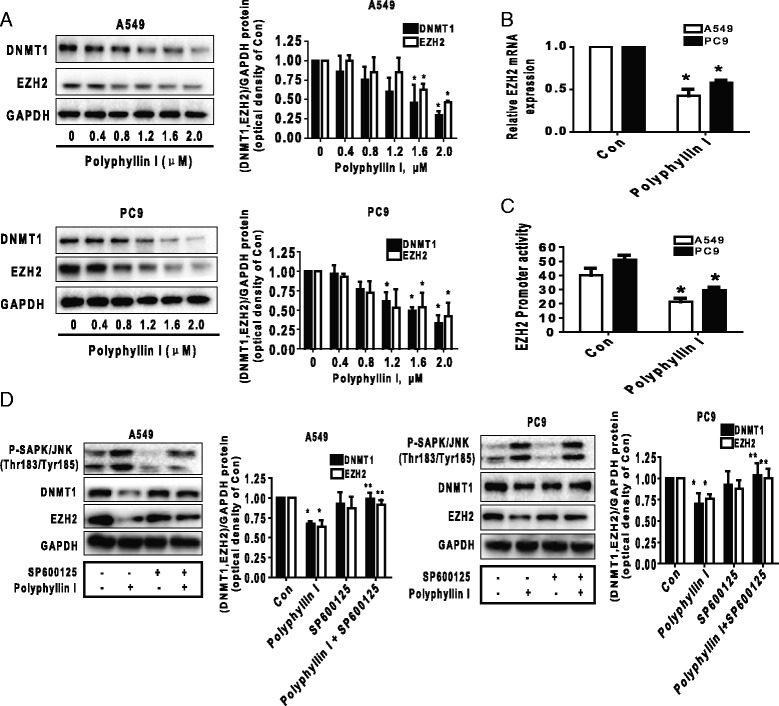
PPI decreased protein expression of DNMT1 and EZH2 through SAPK/JNK pathway. **a** PC9 and A549 cells were exposed to increased concentration of PPI for 24 h. Afterwards, the expression of EZH2 and DNMT1 protein were detected by Western blot. **b** PC9 and A549 cells were exposed to PPI (1.6 μM) for 24 h, followed by measuring the mRNA levels by qRT-PCR. **c** PC9 and A549 cells were transfected with a wild type human EZH2 promoter reporter construct ligated to luciferase reporter gene and internal control for 24 h, followed by treating with PPI for an additional 24 h. Afterwards, the promoter activities were determined using the Secrete-Pair Dual Luminescence Assay Kit as described in the Materials and Methods Section. Values in bar graphs were given as the mean ± SD from three independent experiments performed in triplicate. **d** PC9 and A549 cells were treated with SP600125 (20 μM) for 2 h before exposure of the cells to PPI (1.6 μM) for an additional 24 h. Afterwards, the expression of EZH2 and DNMT1 protein were detected by Western blot using antibodies against EZH2 and DNMT1. The bar graphs represent the mean ± SD of EZH2 or DNMT1/GAPDH of three independent experiments. *Indicates significant difference as compared to the untreated control group (*P* < 0.05). **Indicates significant difference from PPI treated alone (*P* < 0.05)

We also search for the potential molecular targets that may involve in the inhibitory effect of PPI on cell growth. Herein, we showed that PPI decreased DNMT1, the major enzyme responsible for DNA methylation, which is highly expressed in various tumors [35] and EZH2, a key component of polycomb repressive complex 2 (PRC2) and a potential epigenetic silencing of tumor suppressor genes, protein expression levels in a dose-dependent fashion in A549 and PC9 cells (Fig. 3a). Moreover, PPI reduced EZH2 mRNA levels and promoter activity as determined by qRT-PCR and Dual Luminescence assays (Fig. 3b–c) in A549 and PC9 cells. Next, to examine the role of SAPK/JNK signaling in this process, we used specific inhibitors of SAPK/JNK to pre-treated cells. As shown in Fig. 3d, the inhibitor of SAPK/JNK (SP600125) significantly abolished PPI-reduced DNMT1 and EZH2 protein expression levels. This result indicated that activation of SAPK/JNK was involved in the PPI-inhibited protein expression levels of DNMT1 and EZH2.

### Activation of SAPK/JNK pathways-mediated inhibition of p65 expression contributed to the PPI-decreased protein expression levels of DNMT1

**Fig. 4:**
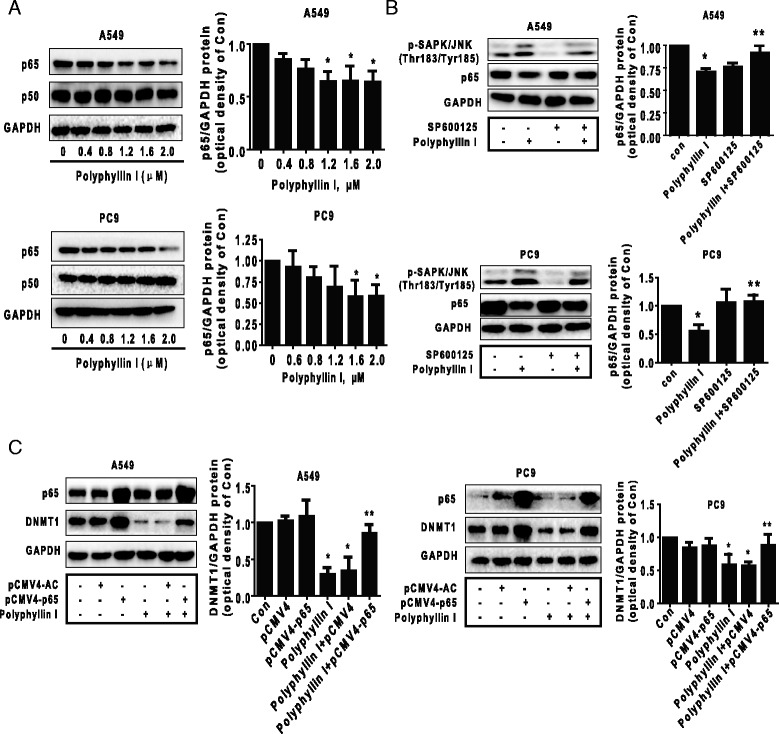
Activation of SAPK/JNK pathway-mediated inhibition of p65 expression contributed to the PPI-decreased protein expression of DNMT1. **a** PC9 and A549 cells were exposed to increased concentration of PPI for 24 h, followed by measuring the protein expression of p65 and p50 by Western blot. The bar graphs represent the mean ± SD of p65/GAPDH of three independent experiments. **b** PC9 and A549 cells were treated with SP600125 (20 μM) for 2 h before exposure of the cells to PPI (1.6 μM) for an additional 24 h. Afterwards, the expression of phosphorylation of SAPK/JNK and p65 protein were detected by Western blot. **c** PC9 and A549 cells were transfected with control and p65 overexpression vector for 24 h before exposing the cells to PPI for an additional 24 h. Afterwards, p65 and DNMT1 protein expressions were determined using Western blot. Values in bar graphs were given as the mean ± SD from three independent experiments performed in triplicate. *Indicates significant difference as compared to the untreated control group (*P* < 0.05). **Indicates significant difference from PPI treated alone (*P* < 0.05)

NF-kB is a key inflammatory transcription factor frequently expressed in cancers. In this study, we further explore the functional relevance of DNMT1 expression after activation of SAPK/JNK. We found that PPI decreased protein expression levels of NF-kB subunit p65, but had no effect on p50 protein (Fig. 4a) suggesting the specificity of PPI. Interestingly, the inhibitor of SAPK/JNK (SP600125) blocked this effect (Fig. 4b). Moreover, exogenously expressed p65 showed to overcame PPI-decreased protein expression levels of DNMT1 and EZH2 (Fig. 4c). Together, the above results indicated that SAPK/JNK signaling pathway was involved in inhibition of p65 protein expression; the latter was contributed to the PPI-decreased protein expressions of DNMT1 and EZH2.

### Exogenously expressed EZH2 not only restored cell growth, but also feedback antagonized PPI-increased SAPK/JNK signaling

**Fig. 5:**
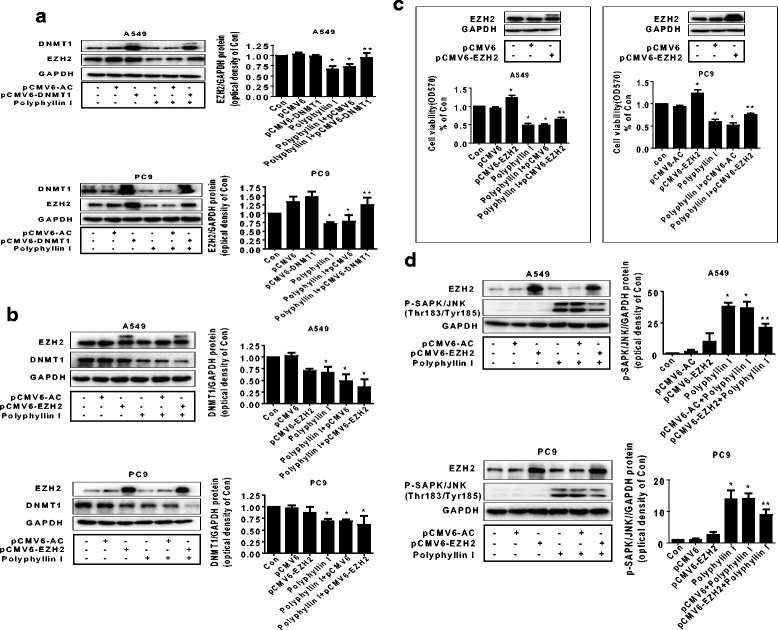
Exogenously expressed EZH2 not only restored cell growth, but also feedback antagonized PPI increased SAPK/JNK signaling. **a** PC9 and A549 cells were transfected with the control or expression constructs of DNMT1 for 24 h before exposing the cells to PPI for an additional 24 h. Afterwards, EZH2 and DNMT1 protein expression were determined using Western blot. **b**–**c** PC9 and A549 cells were transfected with the control or expression constructs of EZH2 for 24 h before exposing the cells to PPI for an additional 24 h. Afterwards, EZH2 and DNMT1 protein expression (**b**) and cell viability (**c**) were determined using Western blot and MTT assays, respectively. **d** PC9 and A549 cells were transfected with the control or expression constructs of EZH2 for 24 h before exposing the cells to PPI for an additional 24 h. Afterwards, EZH2, phosphor-SAPK/JNK were determined using Western blot. Values in bar graphs were given as the mean ± SD from three independent experiments performed in triplicate. *Indicates significant difference as compared to the untreated control group (*P* < 0.05). **Indicates significant difference from PPI treated alone (*P* < 0.05)

To further gain insight into the molecular mechanism by which the interaction of DNMT1 and EZH2 contributed to the overall response of PPI in this setting, we transfected the DNMT1 and EZH2 expression plasmids into the cells, separately. As shown in Fig. 5a–b, overexpression of DNMT1 reversed PPI- decreased EZH2 protein expression levels, on the contrary, exogenous expression of EZH2 had no effect on DNMT1 protein expression. However, exogenous expressed EZH2 could resist PPI-decreased cell growth (Fig. 5c). Intriguingly, it also antagonized PPI-stimulated phosphorylation of SAPK/JNK (Fig. 5d). The above findings suggested the potential interactions of DNMT1 and EZH2, and a negative feedback regulation loop of SAPK/JNK by EZH2 in this process.

### Anti-tumor effects of PPI in xenograft model

**Fig. 6:**
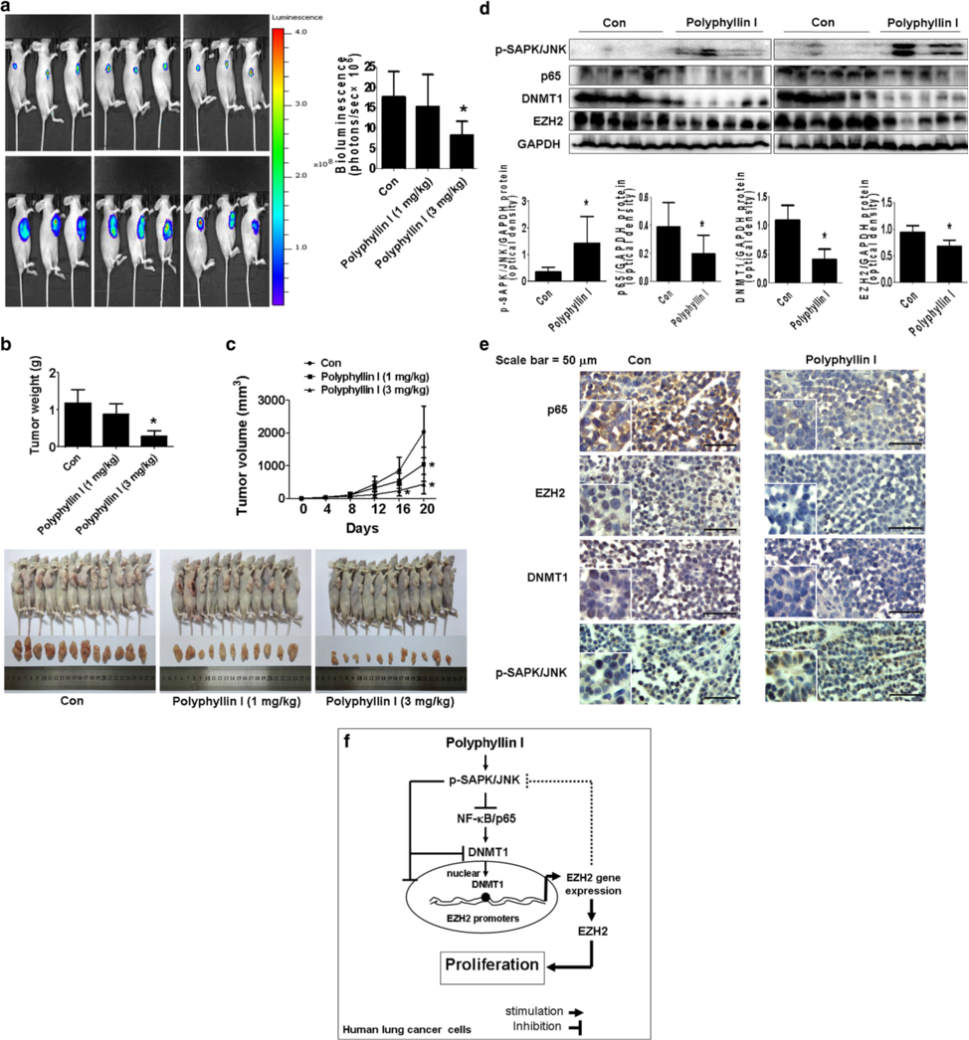
Effects of PPI in subcutaneous xenograft model. Mice (*n* = 12/group) were divided to 3 groups [Con (saline), Low (L, 1 mg/kg) and High (H, 3 mg/kg)], and PPI was given by intraperitoneal injection for up to 27 days. **a** The xenografts were assessed by in vivo bioluminescence imaging at the sixth and end of the experiments (on day 6 and 27). The tumor growth was monitored by injecting luciferin in the mice followed by measuring bioluminescence and analyzed as described in the Materials and Methods section. Representative images are shown. **b** and **c** The xenografts were harvested on day 27, and the size and weight of tumors were determined. The bar graphs represented the tumor weight and size of mice results of as mean ± SD. **d**–**e** At the end of the experiments, xenografted tumors were isolated from individual animals and the corresponding lysates were detected p-SAPK/JNK, DNMT1, p65 and EZH2 proteins by Western blot and Immunohistochemistry as described in the Materials and Methods sections. Scale bar 50 μM. GAPDH was used as loading control for Western blot. Values in bar graphs were given as the mean ± SD from three independent Western blot experiments. *Indicates the significant difference from untreated control (*p* < 0.05). **f** The diagram shows that PPI inhibits growth of NSCLC cells through SAPK/JNK-mediated inhibition of p65 and DNMT1, subsequently; this results in the reduction of EZH2 gene expression. The interactions among p65, DNMT1 and EZH2, and feedback regulation of SAPK/JNK by EZH2 converge on the overall responses of PPI

In order to prove the results in vitro, we tested the effect of PPI in lung cancer xenograft mice model. Mice bearing xenografted lung tumors were treated with control or PPI [8] via intraperitoneal injection for up to 27 days, followed by intraperitoneal injection of D-luciferin. We showed that, compared to the control group, the high doses of (3 mg/kg) PPI-treated mice demonstrated a significant growth-inhibitory effect as assessed by the Xenogen IVIS200 System (Fig. 6a). In addition, we found a significant reduction of the tumor weight and sizes (volume) in the high dose of PPI-treated group as compared to the control group (Fig. 6b–c). Moreover, consistent with the results from the in vitro data, the induced phosphorylation of SAPK/JNK, whereas reduction of p65, DNMT1 and EZH2 protein expression levels from fresh tumors harvested from the aforementioned experiment were observed in the high dose PPI-treatment group as compared to that in the control one using Western Blot and Immunohistochemistry stain (Fig. 6d–e). Note that the low doses of PPI treatment had no significant effects (not shown).

## Discussion

Previous studies showed that natural medicine such as PPI could be considered as a potential agent for the treatment of human cancers. However, the current data are limited and exact mechanisms involving in the anti-cancer activity of PPI have not been well elucidated. In this study, we demonstrated significant cytotoxicity and cell growth inhibition in two NSCLC cell lines confirming the consistence of our findings and further strengthened the hypothesis. This also suggested the possibility of anti-lung tumor activity of PPI. The concentrations of PPI used in this study found to significant inhibit cell viability and induce cell cycle arrest in a dose-dependent manner were consistent, or even low with other studies [3, 7, 8]. Mechanistically, our results indicated that the activation of phosphorylation of SAPK/JNK signaling, regulation and interaction between p65, DNMT1, and EZH2 protein/gene were involved in the anti-lung cancer effect of PPI in this process.

Kinase signaling pathway such as PI3K/AKT/mTOR has been reported to be involved in the PPI-activated autophagy in human hepatocellular carcinoma (HCC) cells [6]. In this study, our results suggested that activation of MAPK family member SAPK/JNK was directly involved in the inhibitory effect of PPI in lung cancer cells. Activation of SAPK/JNK signaling pathway has been reported to regulate the expression of numerous genes associated with apoptosis, invasion, metastasis and survival [21, 36, 37]. We previously found that ursolic acid, a pentacyclic triterpenoid, inhibited growth of NSCLC cells through SAPK/JNK-mediated inhibition of SP1; which in turn resulted in inhibition the protein expression levels of DNMT1 and EZH2 [21]. Fucoxanthin, a marine carotenoid found in brown algae, showed to suppress proliferation and induce cell cycle arrest through SAPK/JNK-mediated induction of DNA damage-inducible protein 45 (GADD45) gene in prostate cancer cells [38]. However, opposite results also have been reported [39], one study showed that blockade of SAPK/JNK signaling was involved in the inhibition of matrix metalloproteinase 9 (MMP9) expression in human gastric cancer cells by a natural compound [40]. Moreover, early study from SAPK/JNK gene deletion experiment in different mouse models demonstrated conflicting results regarding the pro-oncogenic and tumor suppressor roles [41]. Thus, the potential dual roles of this kinase may exist dependent upon the environmental context. More studies, such as siRNAs and overexpression of the constitutive active form of kinase, are needed to characterize the true role of SAPK/JNK in mediating tumor growth.

Our study suggested the involvement of the inhibition of DNMT1 and EZH2 in mediating the effect of PPI on the inhibition of NSCLC cell proliferation. As critical epigenetic and oncogenetic factors, increased expressions of DNMT1 and EZH2 have been shown in several cancer types including lung through silencing tumor suppressor genes via hypermethylation and other mechanisms [42–45]. In addition, expression of DNMT1 and EZH2 were associated with induced proliferation and aggressive metastasis behavior of cancer cells resulting in poor prognosis. Thus, targeting DNMT1 and EZH2 may be of therapeutic benefits for patients with some malignancies [46–48]. Our results also implied the upstream role of SAPK/JNK signaling in the effect of PPI on inhibition of DNMT1 and EZH2. Limited data have been indicated the true links of this kinase signaling to DNMT1 or EZH2 expression or methylation. Early study indicated that the specific inhibitors or siRNA-mediated knockdown of SAPK/JNK decreased DNMT1 expression, thereby inhibiting growth in colon cancer cells [49]. Consistent with ours, one study reported that mitogen-activated protein kinase (MAPK) pathway, such as SAPK/JNK, mediated the down-regulation of EZH2, thereby suppressing growth of breast cancer cells by curcumin [50]. However, another report found that arsenic induced EZH2 phosphorylation through JNK, signal transducer and activator of transcription 3 (STAT3) and Akt signaling pathways in human bronchial epithelial cells [51]. The discrepancy remains unclear; different stimuli, cell lines used and environmental contexts may be accounting for this, which need to be determined with more in-depth experimental approaches. We believed that our findings provided the novel insight into the connection between SAPK/JNK signaling and expression of DNMT1 and EZH2 affected by PPI, and also highlighted the tumor suppressor role of SAPK/JNK that was involved in the anti-tumor effects of PPI.

We also demonstrated a possibility of NF-kB signaling in this process. Our results suggested that inhibition of one of NF-kB subunits p65 protein levels contributed to the PPI-decreased protein expression of DNMT1. One study showed that, nucleolin, an abundant multifunctional phosphoprotein overexpressed in human malignancies, increased DNMT1 gene expression via NF-kB signaling. Inactivation or silencing of NF-kB diminished, whereas exogenous expressed NF-kB increased promoter activity and gene expression of DNMT1 in human leukemia cells demonstrating nucleolin-NF-kB-DNMT1 axis as a new molecular pathway implicated in leukemogenesis [52]. Also, dietary supplements such as curcumin showed to increase G0/G1 cell cycle arrest and induce apoptosis through inhibition of DNMT1 and p65; this resulted in decrease in p65 binding to the DNMT1 promoter implying therapeutic potential of curcumin as an adjuvant treatment in acute myelocytic leukemia [53]. The enhanced NF-kB activity is believed to be highly associated with carcinogenesis. Thus, targeting NF-kB and its relevant signaling may involve in the anti-cancer mechanism of PPI. As EZH2 is also a direct NF-kB target gene [54], whether PPI–inhibited NF-ĸB/p65 signaling had directly effect on EZH2 expression independent of DNMT1 needs to be determined. Nevertheless, more experimental data are required to further elucidate this.

Furthermore, our study suggested that DNMT1 was upstream of EZH2 and that interplay of DNMT1 and EZH2 were responsible for the overall responses of PPI in the inhibition of lung cancer cell growth. The cross-talk and collaborative interaction between these two molecules have been reported in other studies [55, 56], and this may account for some of the oncological properties of EZH2 [29]. One study showed that inhibition of DNMT1 not only decreased EZH2 binding to the promoter regions of cyclin dependent kinase (CDK) inhibitors but also reduced EZH2 expression in human umbilical cord stem cells [57]. Thus, the true role and mechanisms involved in this interaction need to be further elucidated.

Furthermore, we for the first time demonstrated a negative feedback regulation of SAPK/JNK signaling by EZH2 implying that more complicated regulatory loops were involved in the overall effects of PPI. While less data was available for the feedback regulation of SAPK/JNK signaling pathway by EZH2, the potential significance of these regulatory mechanisms involved in the overall responses of PPI required to be determined.

More importantly, our in vivo data were consistent with the findings from that in vitro, confirming the effect of PPI on lung cancer growth inhibition and regulation of SAPK/JNK, p65, DNMT1 and EZH2 protein expression levels. The given doses of PPI were similar with other study demonstrating the substantial anti-tumor effects in the inhibition of human lung cancer [8]. Nevertheless, more experiments are needed to elucidate the precise role of EZH2 in this process using cells stable transfected with shRNA (short hairpin RNA) and expression vectors containing the coding region of EZH2 gene in nude mice model.

## Conclusion

Collectively, our results show that PPI inhibits growth of NSCLC cells through SAPK/JNK-mediated inhibition of p65 and DNMT1 protein expression levels, subsequently; this results in the reduction of EZH2 gene expression. The interactions among p65, DNMT1 and EZH2, and the negative feedback regulation of SAPK/JNK by EZH2 also converge on the overall response of PPI (Fig. 6f). This study reveals a novel mechanism for regulating EZH2 gene expression in response to PPI and suggests a new strategy for NSCLC associated therapy.

## Abbreviations

DMSO, Dimethylsulfoxide; DNMT1, DNA methyltransferase 1; DNMTs, DNA methyltransferases; EZH2, Enhancer of zeste homologue 2; GADD45, DNA damage-inducible protein 45; HCC, Hepatocellular carcinoma; MAPK, Mitogen-activated protein kinase; miRNAs, MicoRNAs; MMP9, Matrix metalloproteinase 9; MTT, 3-(4, 5)-dimethylthiahiazo (-z-y1)-3, 5-di-phenytetrazoliumromide; NF-kB, Nuclear factor-kB; NSCLC, Non-small cell lung cancer; PBS, Phosphate-buffered saline; PI, Propidium iodide; PIK3C2β, Phosphatidylinositol-4-phosphate 3-kinase catalytic subunit type 2 beta; PPI, Polyphyllin I; PRC2, Polycomb repressive complex 2; qRT-PCR, Quantitative real-time PCR; SAPK/JNK, Stress-activated protein kinase/c-Jun N-terminal kinase; SEAP, Secreted alkaline phosphatase; ShRNA, short hairpin RNA; STAT3, Signal transducer and activator of transcription 3; Wnt5A, Wingless-type MMTV integration site family member 5A
